# Supplementary material for: Comprehensive assessment of miniature CRISPR-Cas12f nucleases for gene disruption
Source: Nat Commun. 2022 Sep 24;13:5623. doi: 10.1038/s41467-022-33346-1 (PMC9509373; doi:10.1038/s41467-022-33346-1)
Supplement: Supplementary file 1 — Supplementary Information [file 41467_2022_33346_MOESM1_ESM.pdf]

SUPPLEMENTARY INFORMATION

Comprehensive assessment of miniature CRISPR-Cas12f nucleases for gene disruption

Changchang Xin[1,2], Jianhang Yin[1,2], Shaopeng Yuan[1], Liqiong Ou[1], Mengzhu Liu[1], Weiwei Zhang[1], Jiazhi Hu[1,*]

This PDF file includes:

Supplemental Fig. 1 HEK293T-d2EGFP cells edited by several CRISPR-Cas nucleases

Supplemental Fig. 2 Genome-wide editing off-targets of different Cas nucleases

Supplemental Fig. 3 Cas12f nucleases caused a prevalent deletion accumulated at 2-11bp in length

Supplemental Fig. 4 Repair outcomes and deletional junction′s distribution at *FGF18* locus

Supplemental Fig. 5 The insertion patterns of Cas12f and Cas9 nucleases

Supplemental Table 1 The proportion of GFP negative cells in Cas nuclease-GFP disruption assay

Supplemental Table 2 Bio-primer, Red-primer, and gRNA sequences used in PEM-seq assay

Supplemental Table 3 Vector integrations detected by PEM-seq in this study

Supplementary Fig.1

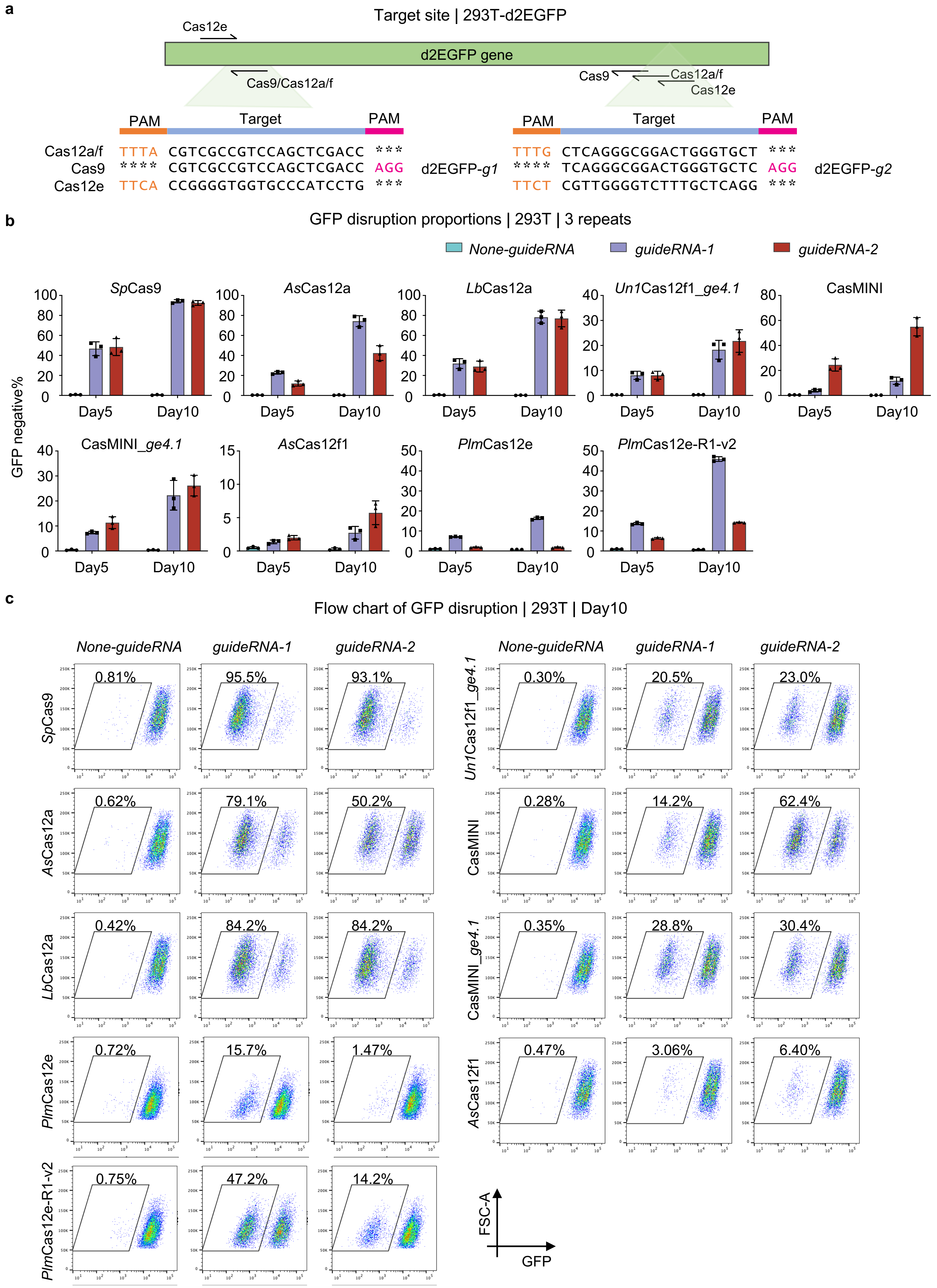

Supplementary Figure 1. HEK293T-d2EGFP cells edited by several CRISPR-Cas nucleases. Related to Figure 1.
(**a**) Map depicting the two target sites by Cas9, Cas12a, Cas12e, and Cas12f Cas nucleases within the EGFP gene for Fig. 1d. (**b**) The GFP disruption efficiencies of different Cas nucleases were shown at the indicated time points and target sites. The GFP disruption proportion is referred to as the number of GFP-negative cells relative to the total number of cells. 'None-guideRNA' represents nontargeting guide RNA. 'guideRNA1' and 'guideRNA2' indicate site 1 and site 2, respectively (N = 3, mean ± SD from three biological replicates). (**c**) Representative raw flow cytometry data by different CRISPR/Cas systems in EGFP disruption assay, with gates showing how the GFP negative cells are gated and numbers above the gates indicating the GFP disruption proportions.

Supplementary Fig.2

a FACS sorting gate
293T | CasMINI-*HBB*

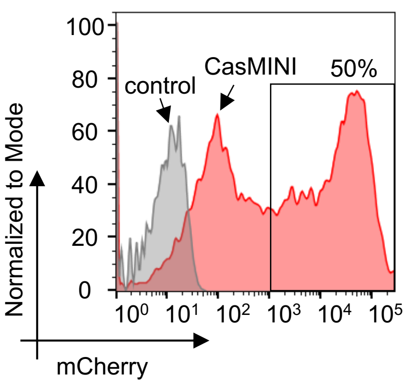

b Editing efficiencies
293T | multiple loci

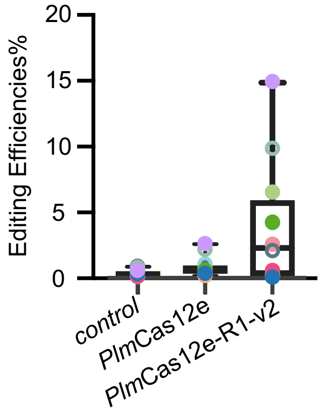

c Off-targets | 293T | multiple loci

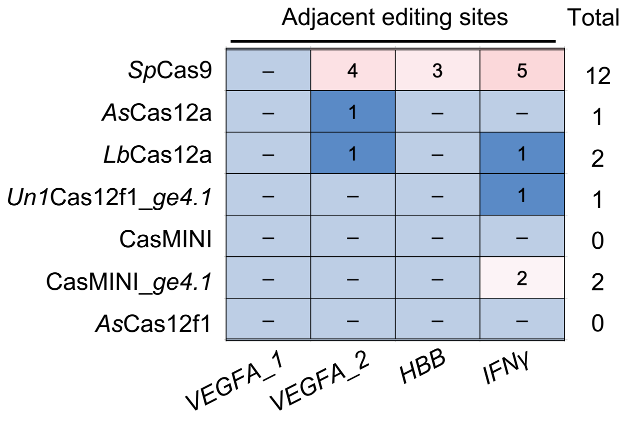

d Off-targets sequencing reads | multiple loci

VEGFA_2

SpCas9

	20 15 10 5 1	PAM	counts
ON	GCTCTCAAGACCCACAATCC	AGG	128467
OT1	TTTCTCAAGACCCACAATTC	AGG	993
OT2	CAACTCAAGACCCACAAGCC	TGG	561
OT3	CACCTCAGGACCCACAATCC	ATG	3
OT4	ACTTTCAAGTCACACAATCC	TGG	3

AsCas12a

	PAM	1 5 10 15 20	counts
ON	TTTG	CTCTCAAGACCCACAATCCA	145370
OT1	TCCT	TTCTCAAGACCCACAATTCA	4

LbCas12a

	PAM	1 5 10 15 20	counts
ON	TTTG	CTCTCAAGACCCACAATCCA	63683
OT1	TCCT	TTCTCAAGACCCACAATTCA	5

NUDT16

SpCas9

	20 15 10 5 1	PAM	counts
ON	GGGGTAGAGGTACTCTACAG	GGG	9163
OT1	GGAGTGGAGGAACTCTACAG	TGG	103
OT2	GGGGTGGAGCCACTCTACAG	AGG	78
OT3	AGGGTAAAGATACTCTAAAG	TGG	75
OT4	AGGGTAGAGGGACTCTCCAG	TGG	23

LbCas12a

	PAM	1 5 10 15 20	counts
ON	TTTA	GGGGTAGAGGTACTCTACAG	74582
OT1	ATGG	AGGGTAAAGATACTCTAAAG	17
OT2	TAGG	GGAGTGGAGGAACTCTACAG	13
OT3	CACT	GGGGTGGAGCCACTCTACAG	8
OT4	TGCA	AGGGTAGAGGGACTCTCCAG	7
OT5	CTTA	GGGGTAGAAGTACTCTAGCA	6

Un1Cas12f1_ge4.1

	PAM	1 5 10 15 20	counts
ON	TTTA	GGGGTAGAGGTACTCTACAG	506575
OT1	GTTA	GGGGTAGGGGTAGGGTTAGG	3

COL8A1

SpCas9

	20 15 10 5 1	PAM	counts
ON	GATTCATTCTCAGTGCCATG	GGG	223518
OT1	GATTCTTTCTCAGTGCCATG	TGG	1796
OT2	TATTCATTATCAGTGCAATG	AGG	3

AsCas12a

	PAM	1 5 10 15 20	counts
ON	TTTA	GATTCATTCTCAGTGCCATG	166787
OT1	ATCA	GATTCTTTCTCAGTGCCATG	11

LbCas12a

	PAM	1 5 10 15 20	counts
ON	TTTA	GATTCATTCTCAGTGCCATG	210421
OT1	ATCA	GATTCTTTCTCAGTGCCATG	6

CasMINI_*ge4.1*

	PAM	1 5 10 15 20	counts
ON	TTTA	GATTCATTCTCAGTGCCATG	346624
OT1	CTTG	TGTTCATTCTCAGTGCCAGC	44
OT2	TTCA	GCTTCATTCTCAGTGCCTGG	60

LNX1

SpCas9

	20 15 10 5 1	PAM	counts
ON	CATACAGGGCTCTGTACCCA	GGG	100432
OT1	GCCACAGGGCACTGTACCCA	AGG	293
OT2	CAAACAGGGCTCTGTACCCA	TGA	8
OT3	TATTCAGGGCTGTGTACCCA	AGG	7

LbCas12a

	PAM	1 5 10 15 20	counts
ON	TTTA	CATACAGGGCTCTGTACCCA	210443
OT1	AGGT	GCCACAGGGCACTGTACCCA	11

Supplementary Figure 2. Genome-wide editing off-targets of different Cas nucleases. Related to Figure 2.
(a) The represented fluorescence-activated cell sorting strategy by CasMINI in *HBB* locus in PEM-seq assay, with gate showing how the plasmid transfected-positive cells are gated for each sample and the number above the gate indicating the cell-sorted proportion. **(b)** Editing efficiency of the control, *Plm*Cas12e and *Plm*Cas12e-R1-v2 at indicated loci detected by PEM-seq. Values from minimum to maximum are shown in the box plot (N = 12). The vertical line through the box is the median. Source data are provided as a Source Data file. (**c**) Number of off-target edits at the adjacent editing sites as detected by PEM-seq methods and identified through the PEM-Q pipeline. '-' means there was no detected off-target site. The indicated locus and Cas nuclease information are marked. (**d**) Sequence alignments and reads counts of PEM-seq detected genome-wide off-targets on *VEGFA_2, COL8A1, NUDT16,* and *LNX1* locus. The off-targets sequence of *Sp*Cas9, *As*Cas12a, *Lb*Cas12a, CasMINI_*ge4.1* and *Un1*Cas12f1_*ge4.1* at indicated locus were listed. Of note, the PAM and off-target counts were marked in the figure, with the translocation junctions within 100 bp from the detected off-target were counted.

Supplementary Fig.3

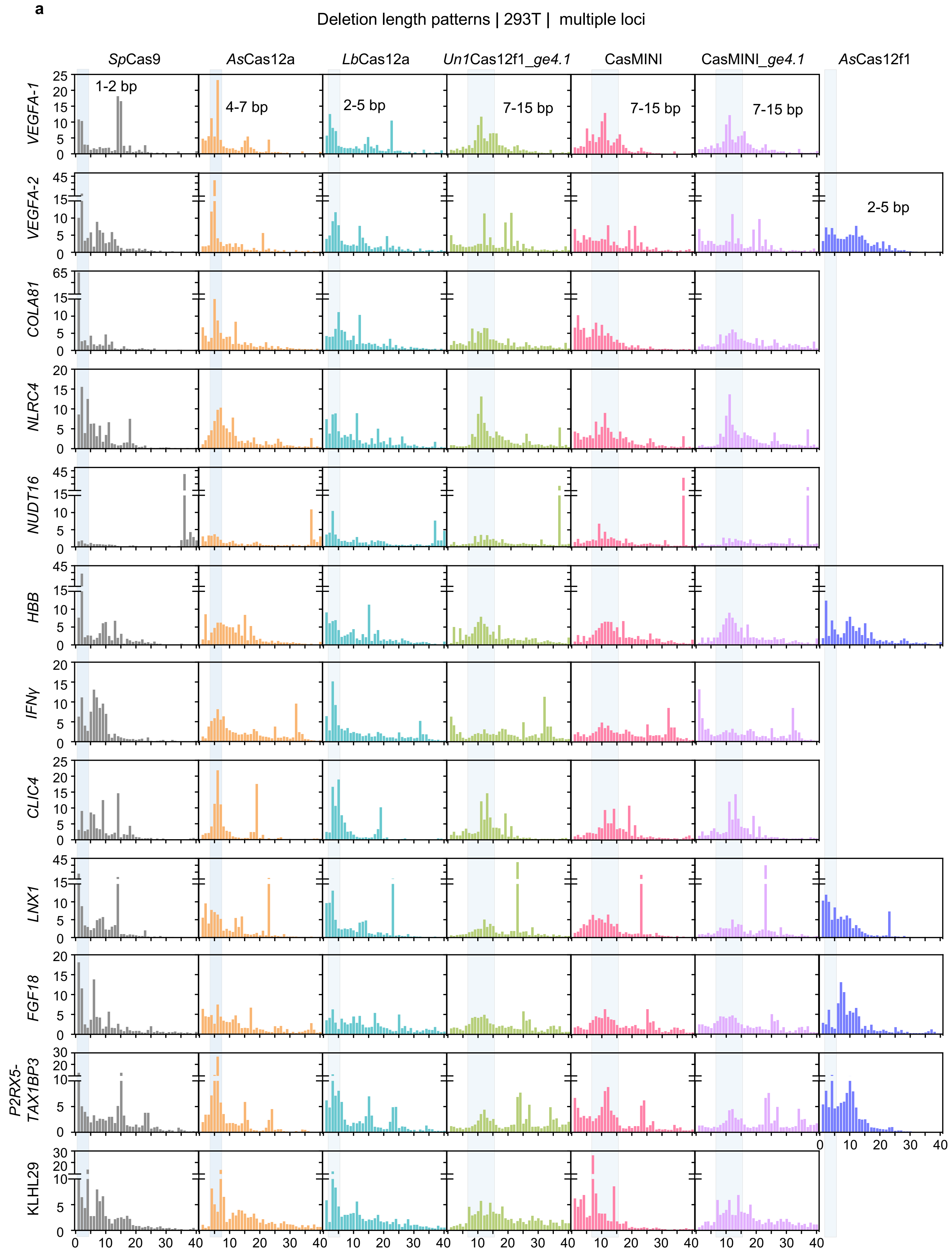

Supplementary Figure 3. Cas12f nucleases caused a prevalent deletion accumulated at 2-11bp in length. Related to Figure 3.

(a) Size and positional information of the deletions, within a length of 40 bp, generated by the indicated Cas nucleases at each indicated tested site. The vertical axis indicates the average ratio refers to the number of deletion fragments with the indicated length to the total number of deletion events. The most abundant deletion size for all tested nucleases is highlighted with a light blue shadow.

Supplementary Fig.4

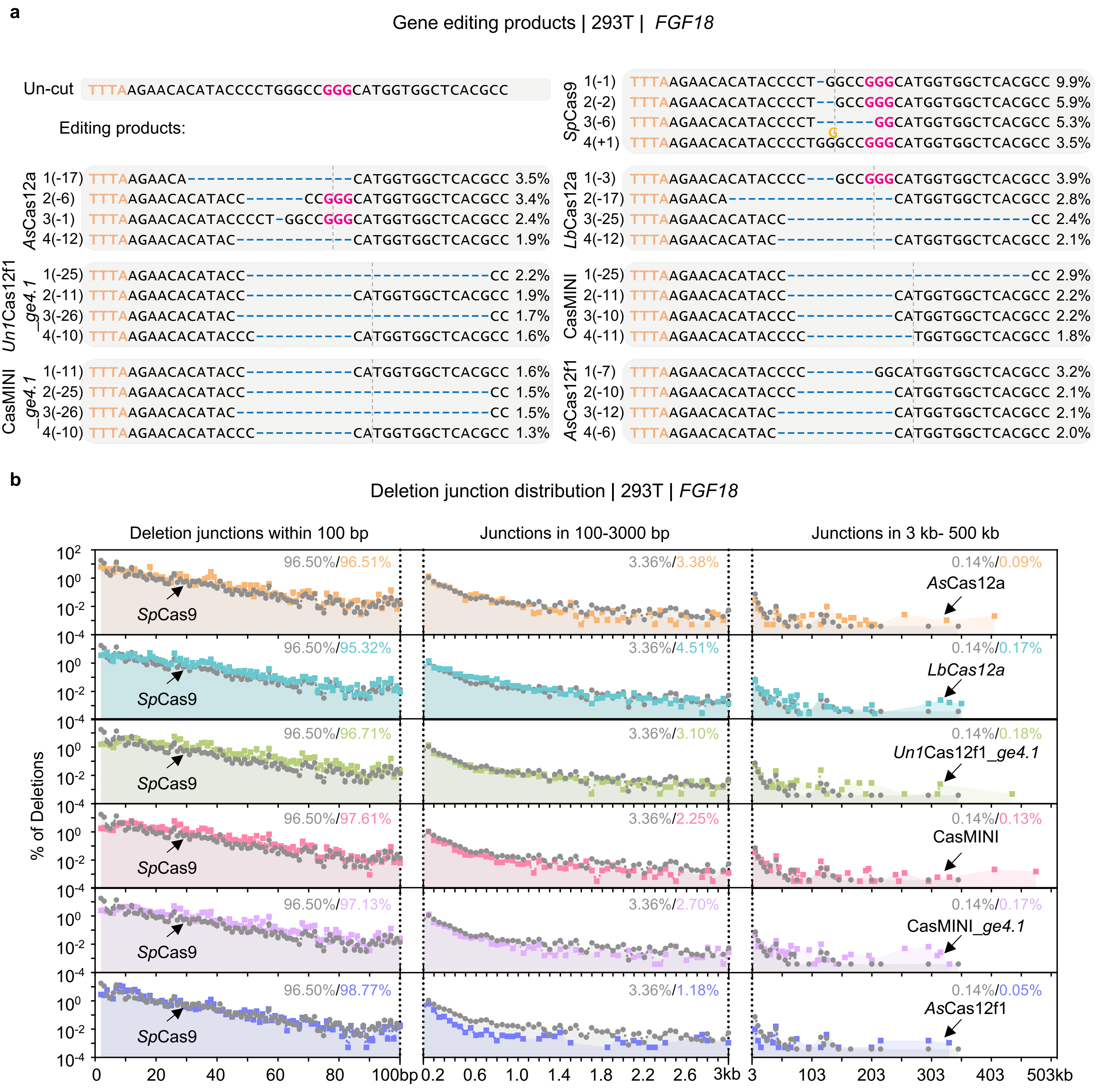

Supplementary Figure 4. Repair outcomes and deletional junction′s distribution at *FGF18* locus. Related to Figure 3. (**a**) Top 4 most abundant editing events for indicated Cas nucleases at the *FGF18* locus detected by PEM-seq in HEK293T cells. The un-cut target sequence was shown on the top with PAM sequence for Cas12 and Cas9 was in orange and fuchsia, respectively. The deleted nucleotides were in dash and the inserted nucleotide were marked with yellow. The cut-sites were marked by the vertical dotted line and the mutation frequencies of indicated editing events were listed on right. (**b**) The deletion junction distribution patterns of indicated Cas nucleases in the *FGF18* locus. Left: junctions within 100bp from the cut site. Medium: junctions in 100bp to 3kb downstream from the cut site. Right: junctions in 3kb to 500kb downstream from the cut site. Please note that 1bp, 50 bp, and 5 kb bin-sizes are used for these three regions, respectively. The number below represents the distance from the cut site. The numbers in different colors at the above right of each box indicated the total proportion of corresponding Cas nuclease′s deletions that occurred in this region.

Supplementary Fig.5

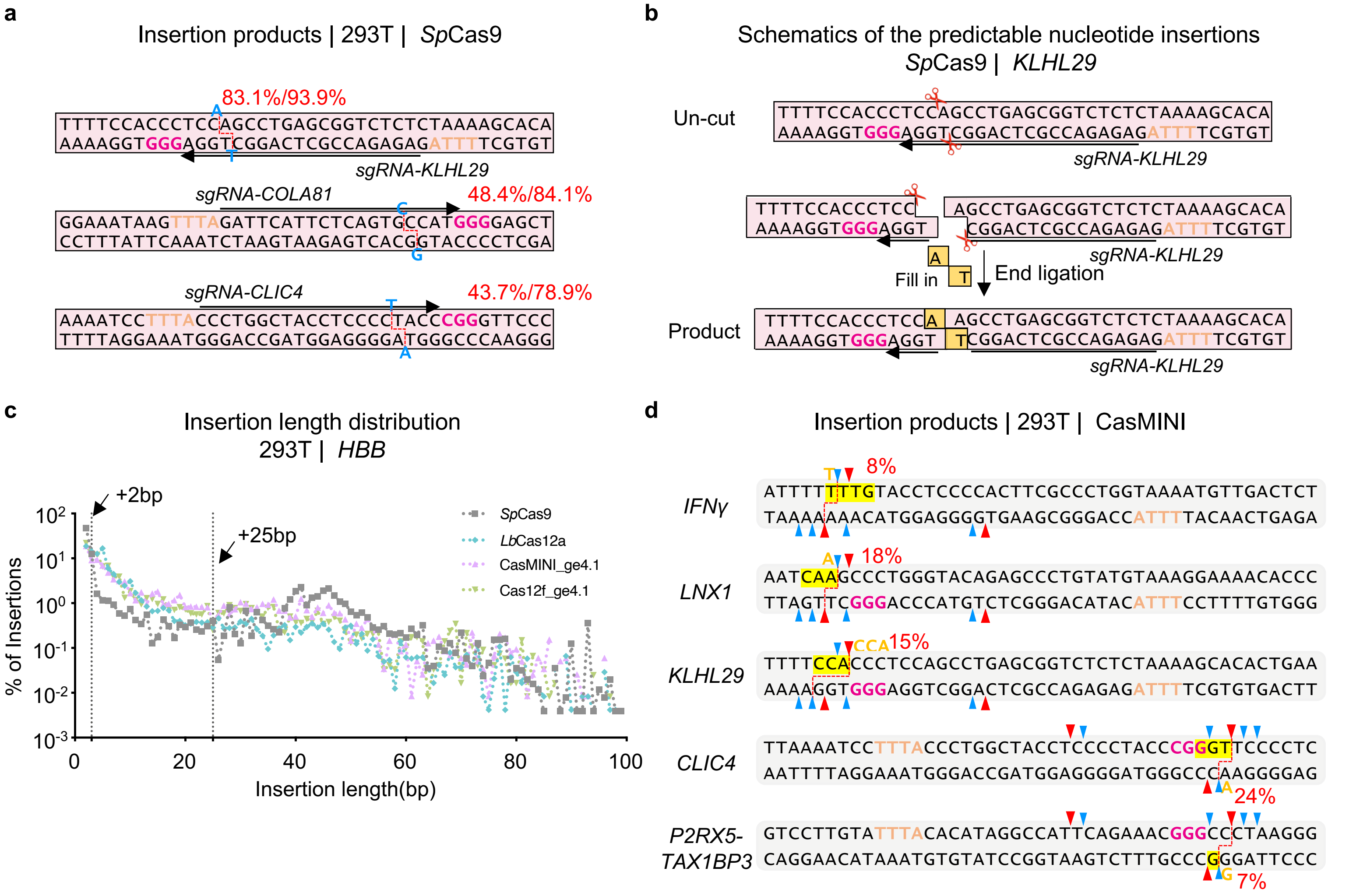

Supplementary Figure 5. The insertion patterns of Cas12f and Cas9 nucleases. Related to Figure 4.
(**a**) The most frequent insertion sequences induced by *Sp*Cas9 at the *KLHL29*, *COL8A1,* and *CLIC4* loci detected by PEM-seq in HEK293T cells with PAM sequences for Cas12 and Cas9 was in orange or fuchsia, respectively. The cut-sites were marked by the red dotted line and the insertion bases were marked in blue. And the mutation frequency of indicated editing events was listed in red with the former and latter numbers indicating the percentage of special insertions relative to total editing events or total insertions, respectively. (**b**) The schematics of the predictable nucleotide insertions at the *KLHL29* locus. The cut-site were marked by the red scissors and the insertion bases were filled with yellow. (**c**) The insertion length distribution with the indicated length at the *HBB* locus in HEK293T cells. Total insertion junctions are plotted on the log scale. The different colors indicate different Cas nucleases, respectively. Black arrows mark 2-bp insertions and 25-bp insertions. (**d**) The most frequent insertion sequences induced by CasMINI at the *IFNγ*, *LNX1*, *KLHL29*, *CLIC4,* and *P2RX5-TAX1BP3* loci detected by PEM-seq in HEK293T cells with PAM sequences for Cas12 and Cas9 in orange and fuchsia, respectively. Red and blue arrowheads indicate the major and minor cleavage sites and the insertion bases were marked in yellow. Then the predicted most frequent blunt end for the PAM-distal segment cut-site was marked by the red dotted line. And the mutation frequency of indicated insertions relative to the total insertions was listed in red.

Supplementary Table 1. The proportion of GFP negative cells in Cas nuclease-GFP disruption assay.

**Cas nucleases**		**Non-target**			**Target site 1**			**Target site 2**		
		R1	R2	R3	R1	R2	R3	R1	R2	R3
*Sp*Cas9	Day5	0.64%	0.98%	0.50%	52.2%	39.1%	49.0%	58.1%	44.5%	42.4%
	Day10	0.41%	0.81%	0.28%	94.8%	95.5%	92.4%	94.4%	93.1%	89.9%
*As*Cas12a	Day5	0.38%	0.53%	0.39%	23.8%	21.3%	23.2%	14.9%	11.3%	9.94%
	Day10	0.18%	0.62%	0.29%	75.5%	79.1%	68.4%	41.8%	50.2%	35.4%
*Lb*Cas12a	Day5	0.38%	0.83%	0.44%	35.5%	26.3%	34.0%	35.2%	25.5%	26.2%
	Day10	0.15%	0.42%	0.28%	78.6%	84.2%	72.2%	79.1%	84.2%	67.9%
Plm Cas12e	Day5	1.07%	1.17%	0.80%	7.24%	6.94%	7.02%	1.88%	1.73%	2.13%
	Day10	0.72%	0.76%	0.72%	15.7%	16.5%	16.8%	1.47%	1.78%	2.09%
*Plm*Cas 12e-R1-v2	Day5	1.10%	0.82%	0.94%	14.3%	13.5%	13.3%	6.40%	6.02%	6.79%
	Day10	0.75%	0.70%	0.52%	47.2%	45.9%	44.9%	14.2%	14.1%	14.5%
*Un1*Cas12 f1_*ge4.1*	Day5	0.30%	0.26%	0.25%	8.83%	9.16%	6.20%	9.34%	8.65%	6.30%
	Day10	0.31%	0.30%	0.36%	20.5%	20.5%	14.0%	25.6%	23.0%	16.8%
CasMINI	Day5	0.45%	0.58%	0.42%	4.12%	4.81%	2.69%	30.1%	22.0%	21.3%
	Day10	0.24%	0.28%	0.28%	12.5%	14.2%	8.56%	55.0%	62.4%	47.8%
CasMINI _*ge4.1*	Day5	0.36%	0.68%	0.26%	7.96%	6.92%	7.29%	13.8%	11.2%	9.10%
	Day10	0.53%	0.35%	0.32%	21.0%	28.8%	17.1%	25.9%	30.4%	22.2%
As Cas12f1	Day5	0.41%	0.68%	0.42%	1.52%	1.54%	1.05%	2.38%	1.88%	1.74%
	Day10	0.14%	0.47%	0.25%	3.49%	3.06%	1.66%	7.09%	6.40%	3.72%

Supplementary Table 2. Bio-primer, Red-primer, and gRNA sequences used in PEM-seq assay.

Site	Bio-primer		Nested-primer	
VEGFA_1	CTTCCCAAAGGACCCCAGTCACTCCAG		GACACACTGTGGCCCCTGTG	
gRNA sequence	Cas9	GCTCTCAAGACCCACAATCC	Cas12a	CTCTCAAGACCCACAATCCA
	Cas12e	GGCCTGGATTGTGGGTCTTG	Cas12f	
VEGFA_2	CCACCCTCTGTCTTATCTCTCCATG		GAAGGGATGTGGTGCATTTGG	
gRNA sequence	Cas9	GCTCTCAAGACCCACAATCC	Cas12a	CTCTCAAGACCCACAATCCA
	Cas12e	GGCCTGGATTGTGGGTCTTG	Cas12f	
COL8A1	CAGAGGAATGGCAAAGCCCTATAAG		GGTCAAGGTTGGAAAGAAGCC	
gRNA sequence	Cas9	GATTCATTCTCAGTGCCATG	Cas12a	GATTCATTCTCAGTGCCATG
	Cas12e	TTCTCAGTGCCATGGGGAGC	Cas12f	
NLRC4	GCAATTGGGCTTATATGCTCCAGGAG		GGCCATTTTGCTTGCCCAATC	
gRNA sequence	Cas9	GAGGGAGACACAAGTTGATA	Cas12a	GAGGGAGACACAAGTTGATA
	Cas12e	CATTTTAGAGGGAGACACAA	Cas12f	
NUDT16	GAGAAGTATAGAAGAGCCAGGTAGG		CCCACAAAGAGAAACCATGTG	
gRNA sequence	Cas9	GGGGTAGAGGTACTCTACAG	Cas12a	GGGGTAGAGGTACTCTACAG
	Cas12e	GGCTTGGGCAAATGAGGCTC	Cas12f	
HBB	GACTTTTATGCCCAGCCCTGGCTCC		GACAGCCGTACCTGTCCTTGG	
gRNA sequence	Cas9	TCCCTCTAAGATATATCTCT	Cas12a	TACTGATGGTATGGGGCCAA
	Cas12e	AACCCTCAGCCCTCCCTCTA	Cas12f	
IFNr	GTGACAGATAGGCAGGGATGATAG		GTGCCATTCTGGTGGGATTC	
gRNA sequence	Cas9	TGTACCTCCCCACTTCGCCC	Cas12a	CCAGGGCGAAGTGGGGAGGT
	Cas12e	CCCTGGTAAAATGTTGACTC	Cas12f	
CLIC4	CCTAACAGGCTACTCCTTCCTGTAG		GGATCAAGGATAGACAAGGTATAG	
gRNA sequence	Cas9	CCCTGGCTACCTCCCCTACC	Cas12a	CCCTGGCTACCTCCCCTACC
	Cas12e	CCTCTCTTTAATTTGGAGAC	Cas12f	
LNX1	GGCCCAGAACCTTGCTCTTTGAG		GGAAATATCCATTGAATTGGCCTG	
gRNA sequence	Cas9	CATACAGGGCTCTGTACCCA	Cas12a	CATACAGGGCTCTGTACCCA
	Cas12e	TTTACATACAGGGCTCTGTA	Cas12f	
FGF18	GAGCTTGGTCAGGGAAGACAGCC		GGACGAAGGATGGGGGAAAGAAG	
gRNA sequence	Cas9	AGAACACATACCCCTGGGCC	Cas12a	AGAACACATACCCCTGGGCC
	Cas12e	TAAAAGCACCCCAGGTGCTC	Cas12f	
P2RX5-TAX1BP3	CAGATTAAATGAAGCGTGAGACAC		CGCCGAGATTTGACTCCTGGAG	
gRNA sequence	Cas9	CACATAGGCCATTCAGAAAC	Cas12a	CACATAGGCCATTCAGAAAC
	Cas12e	GAAACGGGCCCTAAGGGCCT	Cas12f	
KLHL29	GCTACACGCGCTCATTCCTGCCTCC		CAGGATTACTGCAGCACCTCC	
gRNA sequence	Cas9	GAGAGACCGCTCAGGCTGGA	Cas12a	GAGAGACCGCTCAGGCTGGA
	Cas12e	GTGTGCTTTTAGAGAGACCG	Cas12f	

Supplementary Table 3. Vector integrations detected by PEM-seq in this study.

Locus	*Lb*Cas12a	*Sp*Cas9	CasMINI	*As*Cas12a	CasMINI_*ge4.1*	*As*Cas12f1	*Un1*Cas12f1_*ge4.1*
VEGFA_1	12,275	10,478	3,547	5,651	3,336	—	6,545
VEGFA_2	3,588	8,366	1,884	2,560	3,793	390	2,142
COL8A1	2,143	3,070	1,324	2,482	1,249	—	1,526
NLRC4	3,945	6,722	2,318	1,820	1,723	—	3,132
NUDT16	2,948	14,024	1,324	3,946	742	—	631
HBB	1,597	2,807	743	697	965	652	841
IFNγ	3,082	4,748	959	3,254	2,539	—	1,204
CLIC4	1,297	3,386	1,512	2,264	1,199	—	3,015
LNX1	1,024	3,240	493	1,067	1,426	550	1,853
FGF18	964	1,823	1,220	1,156	1,839	912	1,962
P2RX5-TAX1BP3	903	2,386	2,511	1,988	2,224	2,942	1,323
KLHL29	2,230	1,086	755	2,315	813	—	2,530

Each number indicated the vector integration junctions′ number per 100k on-target indels for indicated CRISPR-Cas nuclease at respective locus detected by PEM-seq cloning from the on-target region. K means thousand.
