# Supplementary material for: Comprehensive assessment of miniature CRISPR-Cas12f nucleases for gene disruption
Source: Nat Commun. 2022 Sep 24;13:5623. doi: 10.1038/s41467-022-33346-1 (PMC9509373; doi:10.1038/s41467-022-33346-1)
Supplement: Supplementary file 2 — Reporting Summary [file 41467_2022_33346_MOESM2_ESM.pdf]

nature portfolio

Corresponding author(s): Jiazhi Hu
Last updated by author(s): Sep 5, 2022

Reporting Summary

Nature Portfolio wishes to improve the reproducibility of the work that we publish. This form provides structure for consistency and transparency in reporting. For further information on Nature Portfolio policies, see our Editorial Policies and the Editorial Policy Checklist.

Statistics

For all statistical analyses, confirm that the following items are present in the figure legend, table legend, main text, or Methods section.

n/a	Confirmed	
☐	☒	The exact sample size (n) for each experimental group/condition, given as a discrete number and unit of measurement
☐	☒	A statement on whether measurements were taken from distinct samples or whether the same sample was measured repeatedly
☐	☒	The statistical test(s) used AND whether they are one- or two-sided<br>*Only common tests should be described solely by name; describe more complex techniques in the Methods section.*
☒	☐	A description of all covariates tested
☒	☐	A description of any assumptions or corrections, such as tests of normality and adjustment for multiple comparisons
☒	☐	A full description of the statistical parameters including central tendency (e.g. means) or other basic estimates (e.g. regression coefficient) AND variation (e.g. standard deviation) or associated estimates of uncertainty (e.g. confidence intervals)
☐	☒	For null hypothesis testing, the test statistic (e.g. F, t, r) with confidence intervals, effect sizes, degrees of freedom and P value noted<br>*Give P values as exact values whenever suitable.*
☒	☐	For Bayesian analysis, information on the choice of priors and Markov chain Monte Carlo settings
☒	☐	For hierarchical and complex designs, identification of the appropriate level for tests and full reporting of outcomes
☒	☐	Estimates of effect sizes (e.g. Cohen's d, Pearson's r), indicating how they were calculated

Our web collection on statistics for biologists contains articles on many of the points above.

Software and code

Policy information about availability of computer code

Data collection	Next-generation data were obtained by Illumina Hiseq X-Ten and demutiplexed by Genewiz platform.<br>FACS data were obtained through BD LSRFortessa and BD Sorp.
Data analysis	The supported PEM-seq analysis code - PEMQ pipeline has been uploaded on the GitHub website: https://github.com/liumz93/PEM-Q.<br>FACS data was analyzed by FlowJo (version X 10.4).

For manuscripts utilizing custom algorithms or software that are central to the research but not yet described in published literature, software must be made available to editors and reviewers. We strongly encourage code deposition in a community repository (e.g. GitHub). See the Nature Portfolio guidelines for submitting code & software for further information.

Data

Policy information about availability of data

All manuscripts must include a data availability statement. This statement should provide the following information, where applicable:
- Accession codes, unique identifiers, or web links for publicly available datasets
- A description of any restrictions on data availability
- For clinical datasets or third party data, please ensure that the statement adheres to our policy

The Original PEM-seq sequencing data generated in this study have been deposited in the NODE (National Omics Data Encyclopedia) database with accession code : OEP003371. All plasmids used in this study are available upon reasonable request. Source data are provided in this paper.

Human research participants

Policy information about studies involving human research participants and Sex and Gender in Research.

Reporting on sex and gender	N/A.
Population characteristics	N/A.
Recruitment	N/A.
Ethics oversight	N/A.

Note that full information on the approval of the study protocol must also be provided in the manuscript.

Field-specific reporting

Please select the one below that is the best fit for your research. If you are not sure, read the appropriate sections before making your selection.

[x] Life sciences [ ] Behavioural & social sciences [ ] Ecological, evolutionary & environmental sciences

For a reference copy of the document with all sections, see nature.com/documents/nr-reporting-summary-flat.pdf

Life sciences study design

All studies must disclose on these points even when the disclosure is negative.

Sample size	No sample size calculation was performed. Based on previous studies (Liu et al., 2019, Nature; PMID:30718774), at least three replicates were performed for GFP disruption experiments in cell lies. Besides, at least 3 different loci were performed for PEM-seq experiments in cell lines as previous done (Yin et al., 2022, Nature Communications; PMID: 35260581).
Data exclusions	No data was excluded.
Replication	The EGFP disruption experiments were confirmed by three replicates. For PEM-seq assay, a total of twelve gRNAs were tested independently for each different Cas9 or Cas12 nuclease to confirm the robustness.
Randomization	Randomization is not relevant to the studies. Most samples are prepared from cell lines, sizes are small and the result are consistent.
Blinding	Most analysis are based on PEM-seq. For sample preparation, we need to know the which sample is edited by which nuclease, so no blinding was performed.

Reporting for specific materials, systems and methods

We require information from authors about some types of materials, experimental systems and methods used in many studies. Here, indicate whether each material, system or method listed is relevant to your study. If you are not sure if a list item applies to your research, read the appropriate section before selecting a response.

Materials & experimental systems

n/a	Involved in the study	
[x]	[ ]	Antibodies
[ ]	[x]	Eukaryotic cell lines
[x]	[ ]	Palaeontology and archaeology
[x]	[ ]	Animals and other organisms
[x]	[ ]	Clinical data
[x]	[ ]	Dual use research of concern

Methods

n/a	Involved in the study	
[x]	[ ]	ChIP-seq
[ ]	[x]	Flow cytometry
[x]	[ ]	MRI-based neuroimaging

Eukaryotic cell lines

Policy information about cell lines and Sex and Gender in Research

Cell line source(s)	HEK293T was a gift from Dr. Frederick Alt Lab (Harvard Medical School), which was from ATCC.
Authentication	Cell lines were confirmed by STR (short tandem repeat).
Mycoplasma contamination	Mycoplasma contamination was negative.
Commonly misidentified lines (See ICLAC register)	No

Flow Cytometry

Plots

Confirm that:

- [x] The axis labels state the marker and fluorochrome used (e.g. CD4-FITC).
- [x] The axis scales are clearly visible. Include numbers along axes only for bottom left plot of group (a 'group' is an analysis of identical markers).
- [x] All plots are contour plots with outliers or pseudocolor plots.
- [x] A numerical value for number of cells or percentage (with statistics) is provided.

Methodology

Sample preparation	Cells were isolated with Trypsin followed by centrifugation. PBS were used for washing and cells were resuspended in PBS with 2% FBS followed by FACS analysis.
Instrument	BD LSRFortessa, Aria SORP
Software	FlowJo X (version 10.4), Graphpad prism8
Cell population abundance	For GFP disruption assay, cell population were gated for GFP-negative in HEK293T cells. For PEM-seq sample, cell population were gated for top 30%-40% mCherry-expression in HEK293T cells.
Gating strategy	A lymphocyte gate was defined from FSC-A v SSC-A. Additional GFP gating was executed as described in figure and figure legends for individual experiments.

- [x] Tick this box to confirm that a figure exemplifying the gating strategy is provided in the Supplementary Information.
