# Supplementary material for: Comprehensive assessment of miniature CRISPR-Cas12f nucleases for gene disruption
Source: Nat Commun. 2022 Sep 24;13:5623. doi: 10.1038/s41467-022-33346-1 (PMC9509373; doi:10.1038/s41467-022-33346-1)
Supplement: Supplementary file 4 — Description of Additional Supplementary Files [file 41467_2022_33346_MOESM4_ESM.pdf]

Title: Supplementary Data 1

Description: Statistics of editing events detected by PEM-seq, including the Deletions, Insertions, and Translocations. “Deletion%”, “Insertions%”, and “Translocation%” indicate the ratios of indicated events relative to the total editing events.

Title: Supplementary Data

Description: Red letters indicate the mismatch between the DNA sequences of off-targets and on-targets. “Chr”, chromosome. “Hits” indicates the numbers of translocation junctions within 100 bp from the detected indicated off-targets. “Cleavage%” indicates the ratios of off-target junctions relative to the total on-target indels at the indicated off-target sites. PAM sequences for each site are marked with a horizontal line below.

Title: Supplementary Data 3

Description: “Del”, deletions; “EE”, Editing events; “0-20 bp Del%”, “20-40 bp Del%”, “40-60 bp Del%”, and “60-100bp Del%” indicate the ratios of indicated events relative to the total deletion events.

Title: Supplementary Data 4

Description: “Inser”, Insertions; “EE”, Editing events; “Plasmid Inser/Indel” indicates the ratios of the vector cleavage junctions at the indicated sites relative to the total on-target indels.

Title: Supplementary Data 5

Description: All the DNA sequences of different Cas nucleases and corresponding gRNA sequences are listed, in which the functional elements are marked with indicated colors.
